# Insights on Potential Formation Damage Mechanisms Associated with the Use of Gel Breakers in Hydraulic Fracturing

**DOI:** 10.3390/polym12112722

**Published:** 2020-11-17

**Authors:** Tariq Almubarak, Jun Hong C. Ng, Mohammed AlKhaldi, Saroj Panda, Hisham A. Nasr-El-Din

**Affiliations:** 1 Department of Petroleum Engineering, Texas A&M University, College Station, TX 77843, USA; Cjng@tamu.edu (J.H.C.N.); Hisham.nasreldin@tamu.edu (H.A.N.-E.-D.); 2 Aramco Advanced Research Center—Saudi Aramco, Dhahran 31311, Saudi Arabia; Mohammed.Khaldi.12@aramco.com (M.A.); Saroj.panda@aramco.com (S.P.)

**Keywords:** fracturing fluid, breakers, GPC, oxidizer, enzyme, polymer residue, polymeric clay stabilizer, elemental sulfur

## Abstract

Hydraulic fracturing using water-soluble polymers has been extensively used to enhance the productivity of oil and gas wells. However, the production enhancement can be significantly impaired due to polymer residue generated within the proppant pack in the created fractures. This work describes an approach to establish a suitable fracturing fluid cleanup process by characterizing broken polymer residues generated from the use of different gel breaker types. Commonly used gel breakers such as inorganic oxidizers (bromate and persulfate salts), specific enzymes, and acids were evaluated in this work. The influence of each gel breaker was examined using High-Pressure/High-Temperature (HP/HT) rheometer, aging cells, zeta potential, Gel Permeation Chromatography (GPC), and Environmental Scanning Electron Microscope/Energy Dispersive X-ray Spectroscopy (ESEM/EDS). Experiments were performed on a carboxymethylhydroxypropyl guar (CMHPG) fracturing fluid at temperatures up to 300 °F. The developed GPC methodology showed that the size of the broken polymer chains was mainly dependent on the type of gel breakers used. Moreover, laboratory tests have revealed that some gel breakers may negatively influence the performance of polymeric clay stabilizers. Additionally, this work showed damaging precipitations that can be generated due to the interactions of gel breakers with H_2_S.

## 1. Introduction

Hydraulic fracturing treatments have been used as one of the main stimulation techniques to enhance the productivity of low-permeability oil and gas formations [1,2,3]. These treatments have been applied in relatively shallow to deep hot formations with depth more than 20,000 ft [4,5]. They have also been successfully applied using water salinities up to seawater and even produced water [6,7,8]. One of the key factors that affect the success of hydraulic fracturing treatments is the selection of fracturing fluids and their additives. Optimum fracturing fluids have rheological properties such that it initially provides sufficient viscoelastic properties for fracture initiation and propagation, it is able to suspend proppant into the created fracture, and later it decomposes to a low viscosity fluid at the end of the treatment to allow for fracturing fluid cleanup and hydrocarbon production [9].

There are several fracturing fluids that have been used in fracturing treatments. These fluids can be divided into two main groups, namely oil-based and water-based fracturing fluids. Oil-based fracturing fluids are gasoline gelled with aluminum carboxylates, soaps, viscous refined oils, phosphate esters or gelled crude emulsions [10,11,12,13,14,15,16]. They were used to prevent damage to formations containing water-sensitive clays. With the introduction of clay stabilizers such as potassium chloride, water-based fracturing fluids provided a safer, lower HSE (health, safety, and environment) footprint, and cheaper alternative to oil-based fracturing fluids. Recent developments in clay stabilizer technologies have successfully replaced temporary salt-based clay stabilizers with more permanent polymeric clay stabilizers. Polymeric clay stabilizers are short molecular weight polymers, positively charged, and are able to cover negatively charged clays; preventing them from migrating and inducing formation damage when low salinity water is used.

A typical water-based fracturing fluid contains a polymer thickening agent, clay stabilizer, crosslinker, buffer system, and a gel breaker. Different polymers have been used as thickeners, such as starches and cellulose derivatives [17,18,19]. However, the most common fracturing fluid polymers are guar gum and its two main derivatives hydroxypropyl guar (HPG) and carboxymethyl hydroxypropyl guar (CMHPG) due to their high performance, relatively low price, and wide availability [20,21,22,23,24,25].

Guar gum and its derivatives provide sufficient rheological properties for proppant transport and leak-off control. These rheological characteristics can be reached with a lower concentration of guar gum when crosslinkers are used. There are two main types of crosslinkers: boron-based crosslinkers and metallic crosslinkers and they have been studied extensively by many authors throughout the years [26,27,28,29,30,31,32,33,34,35,36]. These two types of crosslinkers can be used individually or in combination to complement the weaknesses of each other [37,38].

Besides the fracturing fluid rheological properties, its chemical breakage and cleanup characteristics are critical for maintaining a high fracture conductivity. Gel breakers are used to reduce the viscosity of fracturing fluid either by cleaving polymer molecules into smaller fragments or by de-crosslinking the network, which involves removal or rather chelation of the crosslinking molecules [39,40].

Gel breakers can be used in its “live” or encapsulated form. Breaker encapsulation technologies provide some control over the breaker active ingredient release rate. Therefore, the encapsulation provides a delayed activation especially at higher temperature conditions preventing aggressive reactions, early screen outs and corrosion to the tubular [41,42,43,44,45,46]. Due to the many factors that can influence the encapsulated breaker release rate, “live” breakers with no encapsulation were used throughout this work to consistently compare the gel breakers. To break the polymer molecule, three main gel breaker chemical families are typically used in the field: enzymes, oxidizers, and acids [47,48,49].

Enzymes are monomeric or oligomeric proteins that contain hundreds of amino acids. They can fold to form 3D structures that act as catalysts targeting specific bonds in the polymer, causing it to break into smaller fragments, thus, reducing the viscosity of the fracturing fluid. They only target specific chemical bonds in the polymer through a lock and key mechanism, posing a lower risk of side reactions with other additives or the tubular. They are also not consumed by breaking the polymer and will continue to act until they are denatured. Denaturing is the process where the enzyme loses its conformation and shape irreversibly, making it inactive and unable to function, commonly resulting in precipitation. Enzyme denaturing can occur due to several reasons such as high temperature (>150 °F), low or high pH conditions (pH < 4 or pH >10), extreme changes in salt concentrations, presence of solvents and the presence of transitional metals such as iron or zirconium [50,51,52]. Some enzymes have also been known to reduce activity between 8 < pH < 10 or in the presence of calcium chelating agents [53]. However, there are many recent techniques that can be used to enhance the performance of enzymes under the harsh oil and gas field environments. These techniques include the adaptation of enzymes from organisms that live at a much higher temperature, addition of specific ions such as calcium to help strengthen the enzyme structure, enzyme mutation treatments that can enhance the molecular structure (addition of disulfides bridges, increasing hydrogen bonding, increasing internal hydrophobicity), the use of organic additives, and increased pressure to help stabilize the enzyme [54,55,56].

Enzymes typically used to reduce the viscosity of crosslinked polysaccharides are from the *glycoside hydrolase* family. They catalyze the hydrolysis reaction of the *β-1,4* glycosidic bond between the mannose molecules in the polysaccharide backbone to produce simple sugars such as monosaccharides or disaccharides that are soluble in water. Some examples of enzymes used in the oilfield include Amylase, Cellulase, Hemicellulase, Pectinase, and Mannanase [57].

**Figure 1:**
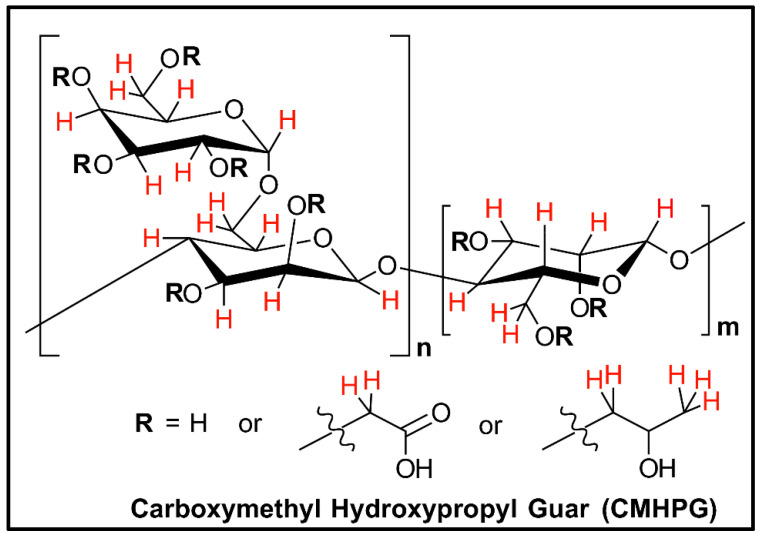
Hydrogen atoms in red are sites on the polymer that can be attacked by oxidizer radical molecules [48].

Oxidizers, on the other hand, work by producing active radicals that can randomly attack hydrogens placed in the polymer structure, as seen with CMHPG in Figure 1. The attack can result in either breaking the mannose backbone into soluble sugars or breaking apart the galactose side chains, producing insoluble residues [48,58].

These radicals are generated at specific temperature conditions. Persulfate salts are typical low-temperature gel breakers (120 °F < T < 200 °F); due to high reactivity at higher temperatures, whereas bromate salts are used for higher temperature applications (T > 200 °F) [49]. Unlike enzymes, the generated radicals are hard to control, limited in quantity, and will get consumed by the different additives in the fracturing fluid. They are also able to react with the tubular, causing corrosion.

Acid gel breakers work in a similar manner to oxidizers; they can break the polymer molecule through hydrolysis reactions in acidic conditions resulting in a variety of insoluble materials. Acid gel breakers can also be used to de-crosslink borate-based fracturing fluids by reducing the amount of monoborate ions (MBI) in solution due to the reduction in pH [59].

Reinicke et al. provided a review of the potential formation and fracture damage processes that result from chemical, physical, and thermal interactions between fracturing fluids and formation components, including fluids and rock constituents [60]. Optimal gel breakers must generate minimum unbroken gel residues to avoid causing any damage to the propped fracture [61].

This paper will provide (1) an overview of typical gel breaker analysis, (2) detailed polymer breakage analysis using Gel Permeation Chromatography (GPC), (3) polymeric clay stabilizer performance analysis in the presence of gel breakers, and (4) gel breaker-induced precipitation in the presence of H_2_S.

## 2. Materials and Methods 

### 2.1. Materials

The fluid used in this work is a 45 lb/1000 gal CMHPG based fracturing fluid. The additives were mixed in the laboratory using typical concentrations used in the oilfield. The fracturing fluid consisted of CMHPG polymer, a high-temperature stabilizer, and a dual crosslinker (borate/zirconium), which were all provided by a service company and used as received, Table 1. Persulfate, bromate, enzyme, and acid “live” gel breakers were supplied by several service companies and were used as received for zeta potential, rheological measurements, GPC, and compatibility experiments. Two polymer clay stabilizers and KCl were supplied by several service companies and used as received for the GPC and zeta potential tests. The compositions of the tested additives are shown in Table 2.

### 2.2. Fluid Preparation

To prepare the 45 lb/1000 gal crosslinked fracturing fluid, 4.32 g of CMHPG was added to 800 mL of tap water (<500 ppm). The fluid was mixed at 400–800 RPM using a blender to create a visible vortex for 20 min. Following that, 7.2 mL of the high-temperature stabilizer was added and mixed at 400–800 RPM for 5 min. Liquid gel breakers were added at this point as required (Acid: 2 gpt or Enzyme: 5 gpt), while solid gel breakers were added directly to the final crosslinked sample in the viscometer (Persulfate: 8 ppt or Bromate: 8 ppt). The pH was adjusted to 10 using NaOH. The crosslinkers were then included by adding 0.64 mL of zirconium crosslinker followed by 0.08 mL of borate crosslinker while mixing at 400–800 RPM. Samples of 52 mL were used for the rheometer tests, and samples of 250 mL were used for the High-Pressure/High-Temperature (HP/HT) aging cell tests that were further used for GPC analysis.

Zeta potential, H_2_S compatibility tests, and specific additive compatibility tests did not include CMHPG or the dual crosslinkers; they were strictly between gel breakers and clay stabilizers, or gel breakers and H_2_S.

### 2.3. Experimental Equipment

#### 2.3.1. HP/HT Rheometer

The HP/HT rheometer was used to measure the apparent viscosity of the gelling samples under different shear rates and temperature ranges. The apparent viscosity is defined as shear stress applied to a fluid divided by the shear rate. The apparent viscosity value for non-newtonian fluids vary depending on the shear rate used. This viscometer utilized a standard R1/B5 bob and rotor combination, which requires a sample volume of 52 cm^3^. The viscometer uses a sliding carbon block for dry heating, and the temperature sensor is mounted on the stator/bob to control sample temperature. A pressure of 1000 psi was applied to prevent the boiling of the samples at high temperatures. Nitrogen gas was used because it is inert towards the fluid.

Viscosity measurements were performed under different shear rates to simulate the flow of the fracturing fluid through production tubular, perforations, and inside the created fracture following the standard ISO13503-1 testing schedule for a duration of 2.5 h [62]. The shear rate range was 100 s^−1^ with varying low shear ramps in the schedule, while the temperature was set at 300 °F. Around 25 mL of fracturing fluid was placed into the rheometer cup, followed by adding solid gel breakers (if required) in the middle, and the remaining 27 mL of the fracturing fluid was then added. This method was used to ensure complete contact between the gel breakers and the fracturing fluid since the quantity of breakers used in each test is extremely low.

#### 2.3.2. HP/HT Aging Cell

The HP/HT aging cells were mainly used to prepare the fracturing fluid samples for gel permeation chromatography analysis. The cells were also used to age the fluids for the zeta potential tests. These experiments were carried out in 250 mL glass bottles that were inserted to the HP/HT cells, pressurized by nitrogen to 400 psi and heated to 200–300 °F for 0.5–48 h.

#### 2.3.3. Gel Permeation Chromatography

**Figure 2:**
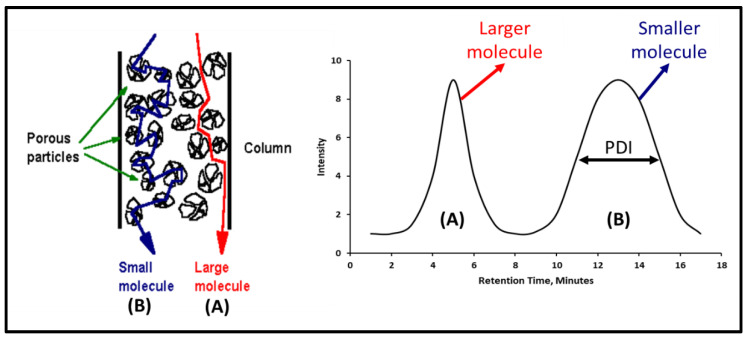
Simplified Gel Permeation Chromatography (GPC) illustration and example showing that molecule A size > molecule B size, and molecule B PDI > molecule A PDI.

A simplified representation of a GPC column and its corresponding results is shown in Figure 2. An analysis is typically done using known molecular weight standards to determine the peak molecular weight of polymer fragments being measured. Jackson et al. showed that peak molecular weight (Mp) is a representation of the most abundant molecular weight in the sample [63]. A curve fit can be done with the calibration points to extend the range beyond the standards being used. However, these values would not be as accurate as the values within the known standard values. For that reason, only values report within the sizes of the standards used are reported in this work.

The developed aqueous gel permeation chromatography (GPC) method was applied to determine the Mp and the distribution profile of crosslinked 45 lb/1000 gal polymer samples treated with gel breakers. About 150 mg of each sample was dissolved in 5 mL of 0.05 M NaNO_3_. The diluted samples were shaken with a mixer at 300 rpm for 30 min. Then, the samples were directly injected into the GPC system. The standard solutions for calibration of the GPC column were prepared by dissolving 2 mg of each pullulan standard in 1 mL of 0.05 M NaNO_3_ in separate vials. The standard solutions were dissolved for 1 h to achieve complete dissolution of the polymer with occasional shaking. Eight pullulan calibration standards with the following peak molecular weights (Mp) values: 21,100; 47,100; 107,000; 194,000; 344,000; 708,000; 1,220,000 and 2,350,000 g/mol were used for the calibration of the column. All the GPC separations were carried out on a PL aquagel-OH MIXED-H (7.5 × 300 mm, 8 µm) column. The mobile phase was 0.05 M NaNO_3_. The flowrate throughout the separation was maintained at 0.8 mL/min.

A separate aqueous GPC method was developed to determine the peak molecular weight (Mp) and the distribution profile of heated and gel breaker-treated clay stabilizer polymer samples. All the treated samples were filtered through a 0.45 µm hydrophilic syringe filter before the analysis with GPC. Polyethylene glycol standards with a peak molecular weight (Mp) 106; 194; 282; 420; 610; 1010; 1480; 4040; 7830; 16,100; 21,300, 34,890, 47,100; 107,000; 194,000 and 344,000 g/mol were used for the calibration of the column. All samples were separated on a PSS NOVEMA Max (300 × 8 mm, 10 µ) 100 Å columns. The mobile phase was 0.05% formic acid, with a maintained flowrate of 0.8 mL/min.

All GPC separations for both sets of samples were carried out on an Agilent 1260 series high-performance liquid chromatography with a binary pump, a degasser, an auto-sampler, and a refractive index detector. Instrument control and GPC data analysis were performed through the OpenLAB and Cirrus software, respectively.

#### 2.3.4. Zeta Potential

The zeta potential was measured by using micro electrophoresis. Changes in zeta potential were measured for illite crushed samples in solutions containing different clay stabilizers. Each sample was prepared by weighing out 1 g of illite particles suspended in a total of 250 mL of DI water (resistivity of 18 Ω.cm). Each of these suspensions were ultrasonicated for 30 min in a sonic bath. Following that, clay stabilizers and gel breakers were added to the samples (Persulfate: 8 ppt; Bromate: 8 ppt; Acid: 2 gpt; Enzyme: 5 gpt), shaken by hand, and aged for 24 h at 200–300 °F using aging cells as required. After aging, the samples were shaken by hand and stood at room temperature (77 °F) for another 24 h to reach equilibrium prior to analysis. The supernatant was then used for analysis.

The used electrode assembly was conditioned in 1 M NaCl using 350 cycles. This conditioning procedure produces a uniform black coating on the electrodes, which is vital for zeta potential analysis of suspensions in high ionic strength solutions.

The particle suspension was added as required to the cuvette at a 45° angle to avoid trapping air bubbles between the electrodes. Visual inspection for bubbles on the surface of the cuvette or between the electrodes is required to ensure proper measurements. Air bubbles can often be dislodged by gently tapping on a hard surface. The cuvette was then placed in the instrument and allowed to equilibrate to the measurement temperature of 77 °F for a period of 5 min.

#### 2.3.5. Sour Environment Compatibility Tests

**Figure 3:**
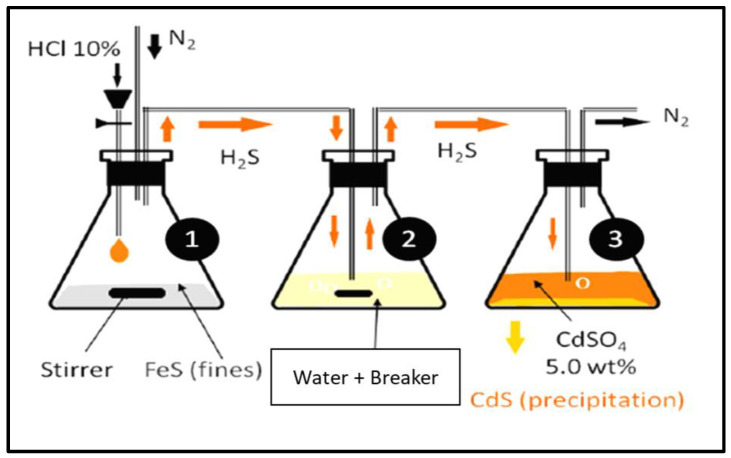
Sour environment compatibility experiment setup.

Several tests were conducted to investigate the interaction of gel breakers with H_2_S at 77 °F. The H_2_S compatibility tests were conducted using a closed system loop. H_2_S was generated by reacting 1 g of iron sulfide (FeS) with 10 mL of 10 wt% HCl in an Erlenmeyer flask, the generated H_2_S gas was then diverted to a second flask containing a solution of 200 mL of DI water (resistivity of 18 Ω.cm) and the gel breaker at the desired concentration (Persulfate: 8 ppt; Bromate: 8 ppt; Acid: 2 gpt; Enzyme: 5 gpt). After exposing the solution to H_2_S gas, the H_2_S was then diverted to a third flask containing 200 mL of 5 wt.% of cadmium sulfate (CdSO_4_), where the unreacted H_2_S was scavenged completely into solid cadmium sulfide (CdS). The setup is shown in Figure 3. The H_2_S experiments lasted 4 h, and the middle flask solution was then filtered through 0.2 µm filter paper and washed with DI water to determine if there was any precipitation. The same experimental procedure was conducted several times to assess the interaction of H_2_S with typical fracturing fluid gel breakers as a function of the gel breaker type.

#### 2.3.6. Environmental Scanning Electron Microscope

The environmental scanning electron microscope (ESEM) with integrated ultra-thin window energy dispersive X-ray detector was utilized to perform comprehensive compositional characterizations of precipitations resulting from gel breaker interactions in sour environments. The samples were first prepared by filtering the precipitation out of solution using a 0.2 µm filter paper. After that, the samples were dried at 140 °F in the oven for 24 h. The Environmental Scanning Electron Microscope/Energy Dispersive X-ray Spectroscopy (ESEM/EDS) data are required to identify the minerals in the sample.

## 3. Results and Discussion

### 3.1. Fracturing Fluid Viscosity Tests

**Figure 4:**
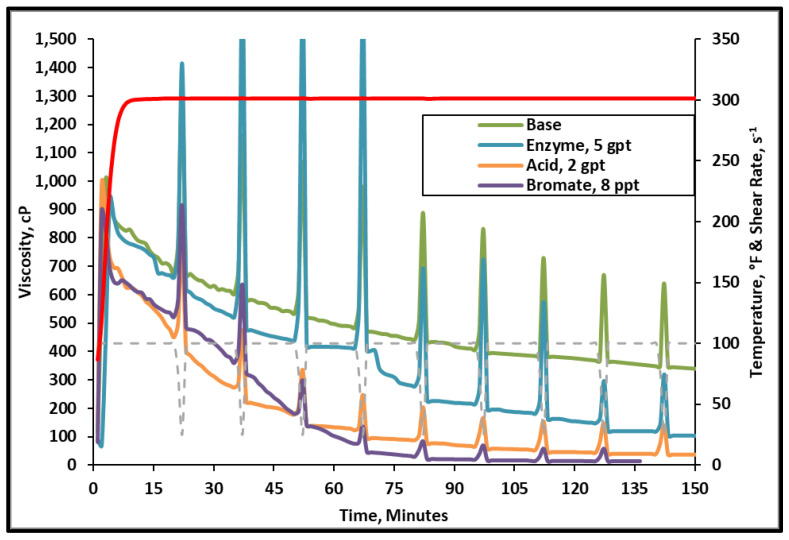
Viscosity of 45 lb/1000gal crosslinked fracturing fluid as a function of gel breaker type at 300 °F.

**Figure 5:**
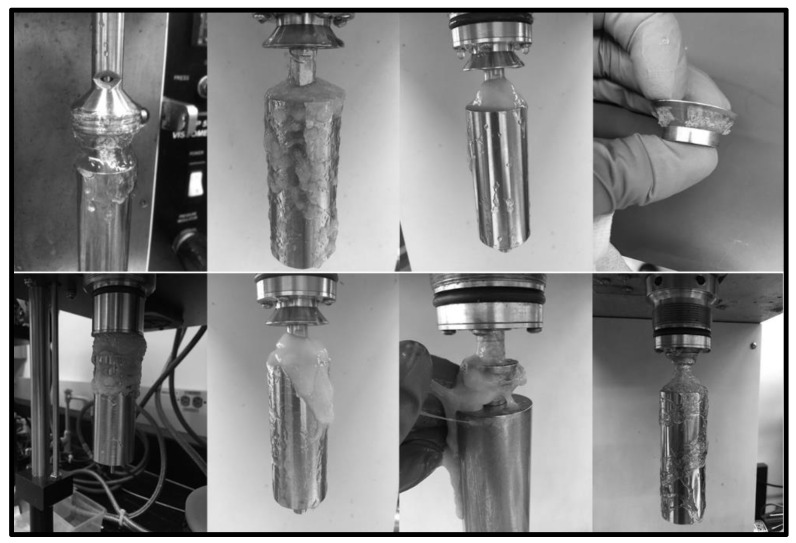
Unbroken polymer residue remaining after various breaker evaluation tests at 300 °F (oxidizers and acids), yet they still exhibited low final viscosity (<50 cp @ 100 s^−1^).

The fracturing fluid and gel breaker viscosity were first measured to determine the general influence of different gel breakers at 300 °F. The viscosity of the fracturing fluid without any gel breaker was averaging 350 cP after 2 h at 300 °F and 100 s^−1^ shear rate. The fracturing fluid viscosity after 2.5 h at the same temperature with bromate, acid, and enzyme gel breakers were 20, 50, 130 cP, respectively, Figure 4. This test shows that all gel breakers are effective in breaking this fracturing fluid. Although the fracturing fluids containing bromate or acid were low in final broken viscosity values, when the fluids were taken out from the viscometer, the remaining effluents contained portions of the crosslinked fluid that were left unbroken. This has also been observed in several previous gel breaker evaluation tests that exhibited low viscosity yet still contained a significant amount of unbroken fracturing fluid residue, Figure 5. Further tests were conducted using GPC to understand the influence of each breaker type.

### 3.2. Fracturing Fluid Polymer-Gel Breaker GPC Analysis

Several samples of the high temperature fracturing fluid were aged using HP/HT cell for 0.5, 2, 4, and 24 h at 200 °F with persulfate and at 300 °F with bromate, acid, and enzymes. Visually, in the absence of gel breakers, the crosslinked samples remained crosslinked and showed no obvious reduction in the viscosity upon tilting after 24 h. The samples that contained gel breakers showed a significant visual reduction in viscosity at all tested time periods.

**Figure 6:**
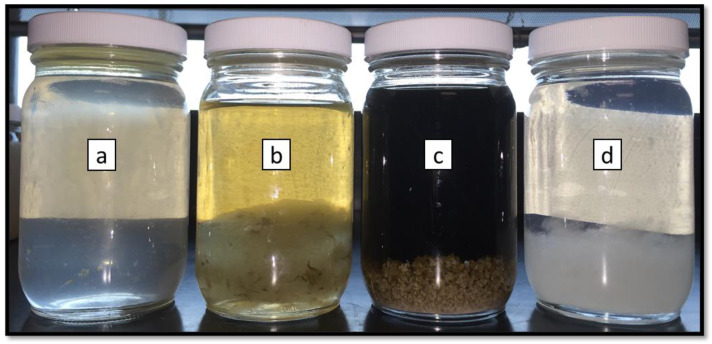
Residual polymer in the broken crosslinked fracturing fluid samples after exposure to different gel breakers ((**a**): enzyme—5 gpt, (**b**): acid—2 gpt, (**c**): bromate–8 ppt, (**d**): persulfate—8 ppt) after 24 h at 200–300 °F.

After the crosslinker fracturing fluids were broken by the gel breakers, the samples were visually inspected for polymer residue. The fracturing fluid samples in the presence of bromate, persulfate or acid gel breaker that were tested for 24 h were not homogenous. Clumps of polymer were separated from the less viscous water phase. The degree of residue and separation was highest in the samples containing acid gel breakers and the least in the samples containing enzyme gel breaker, Figure 6.

**Figure 7:**
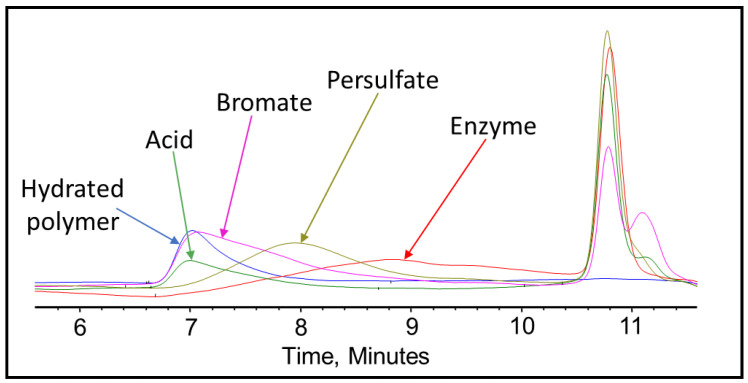
GPC results of the 45 lb/1000 gal crosslinked fracturing fluid exposed to different gel breakers (persulfate: 8 ppt, bromate: 8 ppt, acid: 2 gpt, enzyme: 5 gpt) after 0.5 h at 200–300 °F.

**Figure 8:**
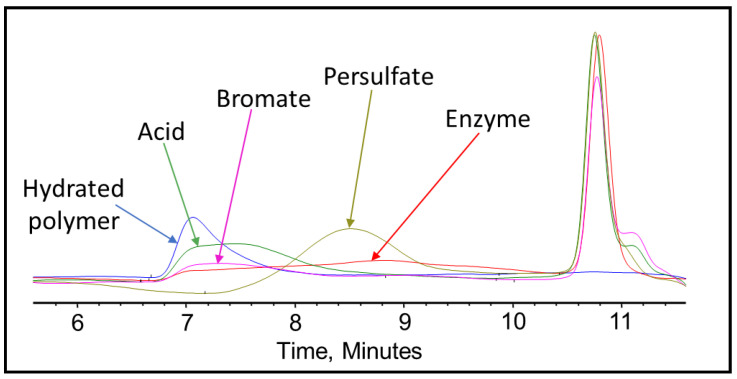
GPC results of the 45 lb/1000 gal crosslinked fracturing fluid exposed to different gel breakers (persulfate: 8 ppt, bromate: 8 ppt, acid: 2 gpt, enzyme: 5 gpt) after 2 h at 200–300 °F.

**Figure 9:**
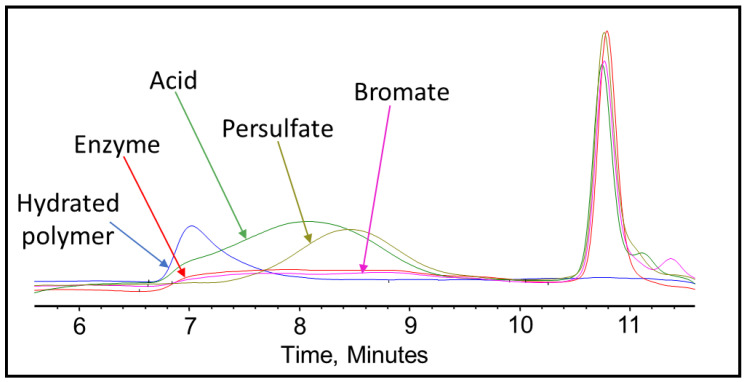
GPC results of the 45 lb/1000 gal crosslinked fracturing fluid exposed to different gel breakers (persulfate: 8 ppt, bromate: 8 ppt, acid: 2 gpt, enzyme: 5 gpt) after 4 h at 200–300 °F.

**Figure 10:**
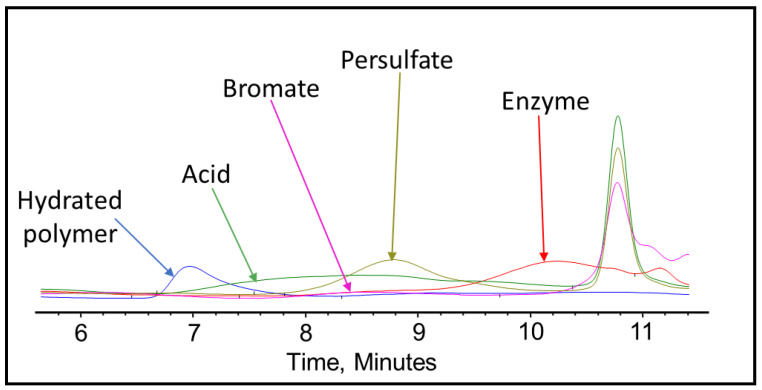
GPC results of the 45 lb/1000 gal crosslinked fracturing fluid exposed to different gel breakers (persulfate: 8 ppt, bromate: 8 ppt, acid: 2 gpt, enzyme: 5 gpt) after 24 h at 200–300 °F.

After completing the visual inspection, the samples were tested thoroughly using GPC to analyze and compare the effect of persulfate, bromate, acid, and enzyme gel breakers on the fracturing fluid polymer. The goal of this test was to compare the approximate broken polymer size in all gel breaker solutions at different time intervals. The peak molecular weight size in different gel breaker treated samples at 4 h were from highest to lowest: enzyme, acid, persulfate, bromate, Figure 7, Figure 8 and Figure 9. The samples that were aged for 24 h showed that the peak molecular weight comparison from highest to lowest was the following: acid, bromate, persulfate, enzyme, Figure 10.

**Figure 11:**
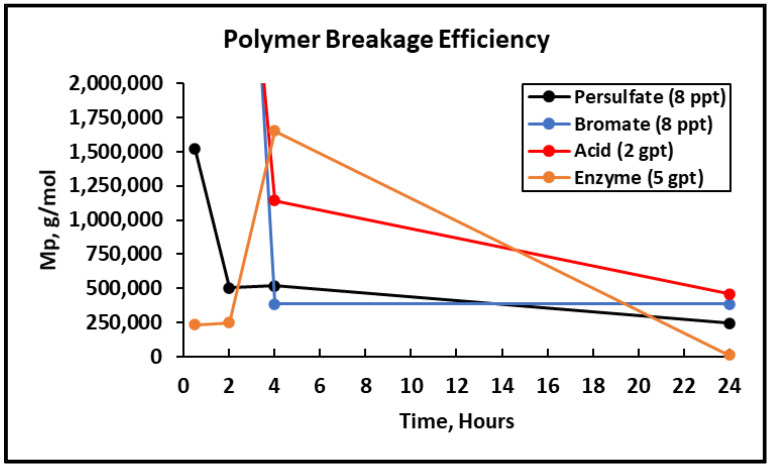
Peak molecular weight values for the 45 lb/1000 gal crosslinked fluid mixed with gel breakers at different time intervals at 200–300 °F.

It was noted that the bromate gel breaker reactions stopped after around 4 h, as the molecular weight of the broken crosslinked polymer did not change noticeably. The persulfate and acid breakers continued performing after 4 h but did not produce the smallest molecular weights. However, the enzyme was fully functional after 24 h and contributed to the lowest final peak molecular weight compared to all other gel breakers, Figure 11 and Table 3.

It was also noted that for the enzyme-treated sample, the peak molecular weight has shifted to a larger molecular range at 4 h of the gel breaker treatment. This could be due to the method of enzyme attack. The enzyme attaches to a polymer strand while cleaving the glycosylic bonds and does not leave until the polymer is completely broken [48]. Therefore, in this case, the enzymes could have broken many long-chain polymers initially, which resulted in low peak molecular weight values at short aging periods. After 4 h, the initially attacked polymers would have been broken into a variety of sizes, leaving the majority of polymer remaining to be long-chained and relatively unbroken polymers. By the end of the 24 h, the enzyme would have the time to break all the chains relatively equally into much smaller parts, which resulted in a lower peak molecular weight compared to the other gel breakers.

### 3.3. Zeta Potential

Zeta potential experiments were conducted to assess the fines migration tendency of illite clays in different solutions. Several experiments were initially conducted with and without the presence of clay stabilizers to determine their effect on illite clay particles zeta potential value in distilled water. Similar experiments were conducted in the presence of gel breakers at 77–300 °F. These experiments were mainly performed to determine the effect of different gel breakers on the performance of clay stabilizers.

**Figure 12:**
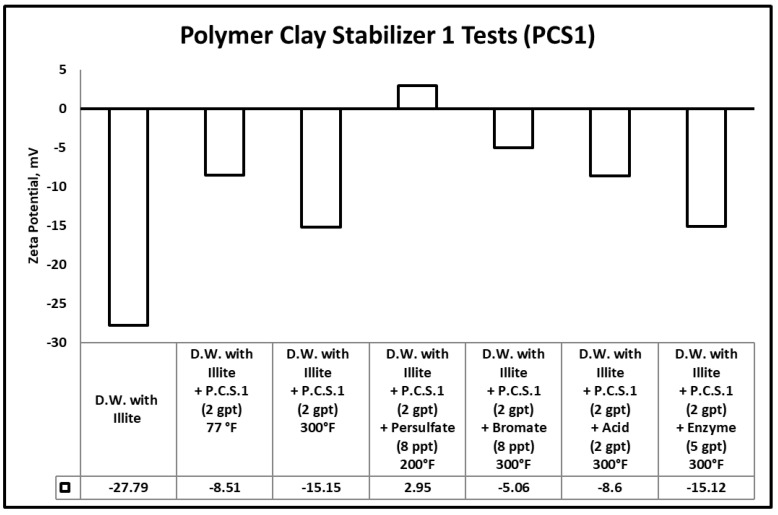
Zeta potential results for the tests involving gel breakers and polymeric clay stabilizer 1.

**Figure 13:**
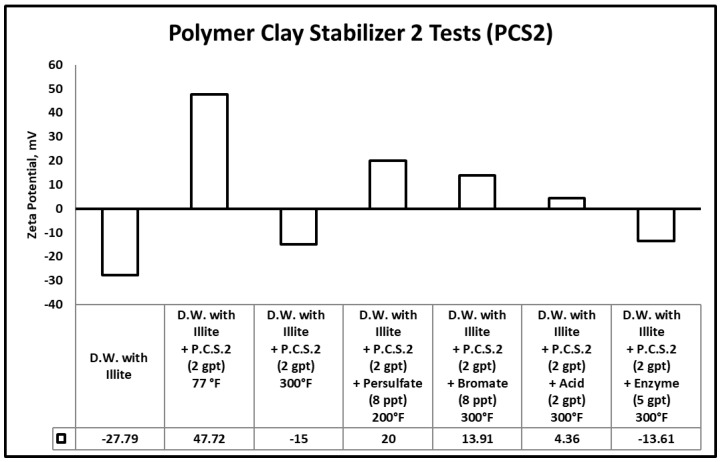
Zeta potential results for the tests involving gel breakers and polymeric clay stabilizer 2.

**Figure 14:**
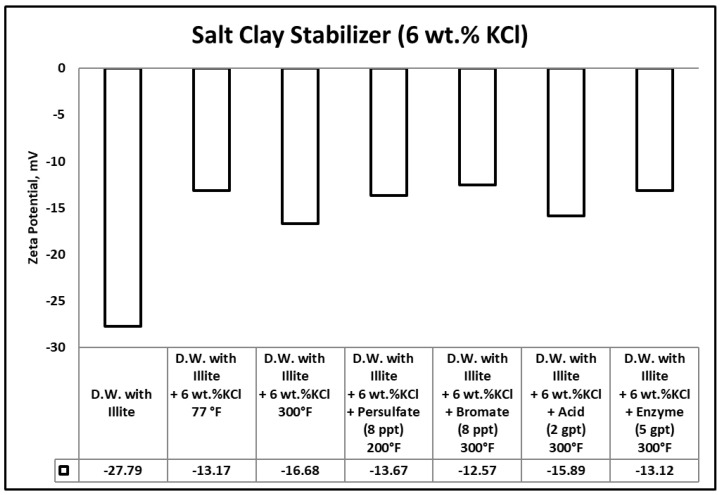
Zeta potential results for the tests involving gel breakers and KCl clay stabilizer.

The potential for fines migration is mainly dependent on the changes in the clay edge and surface charges. Measured zeta potential of any clay reflects the combined value for the surface and edge charge potentials [64]. When zeta potential becomes highly negative or positive (ζ-potential < −20 or ζ-potential > 20), it produces a significant repulsive force with similarly charged particles in solution. Sandstone formation rocks are negatively charged and having a highly negative zeta potential value causes colloidal induced detachment of fines [65,66]. In other words, this highly negative value of zeta potential will increase the repulsive colloidal forces, which triggers the fines migration cases. Figure 12, Figure 13 and Figure 14 summarize the zeta potential results for solutions of illite in different clay stabilizer and gel breaker concentrations at temperatures between 77–300 °F.

The zeta potential value in the samples containing illite fine particles suspended in distilled water at room temperature (77 °F) was −27.79 mV. This indicates the instability of the clays and the possibility of fines migration damage in the formation. The zeta potential values in samples containing illite fine particles suspended in distilled water with polymeric clay stabilizer 1, polymeric clay stabilizer 2 and 6 wt.% KCl at room temperature (77 °F) were −8.51, 47.72, −13.17 mV, respectively. The values have increased compared to the base case where no clay stabilizer was used, indicating higher clay stability in the presence of either of the clay stabilizers. Comparatively, the results at 300 °F with polymeric clay stabilizer 1, polymeric clay stabilizer 2, and 6 wt.% KCl were −15.5, −15, −16.68 mV. This shows that temperature does negatively influence both polymeric clay stabilizer 1 and 2.

Clay stabilizer 1 and 2 are polymeric clay stabilizers containing positive charges that allow them to attach and cover the different clays protecting them from exposure to fluids that can cause them to swell or migrate. Clay stabilizer 2 and illite solution zeta potential value was highly positive, providing attraction between these positively coated clays and the uncoated negatively charged clays, indicating that it is less likely to have chemically induced fines migrations. The difference between clay stabilizer 1 and 2 is the composition and the molecular weight. Clay stabilizer 2 had a significantly higher molecular weight compared to clay stabilizer 1, producing more charges and producing the difference in zeta potential results.

The zeta potential values of several samples were also measured after the addition of persulfate, bromate, acid, and enzyme gel breakers to solution of clay stabilizers and illite. The goal of the test was to check if there are any interactions between the gel breakers and polymeric clay stabilizers to determine if the polymeric clay stabilizers are still able to prevent clay problems during the treatment.

The zeta potential values of the samples containing illite fine particles suspended in a solution of distilled water and polymeric clay stabilizer 1 with persulfate, bromate, acid, or enzyme gel breakers after aging at 200–300 °F were 2.95, −5.06, −8.6 and −15.12 mV, respectively. These results show that the polymeric clay stabilizer 1 zeta potential values did not change significantly after exposure to the enzyme compared to the base case at 300 °F, which had a value of −15.15 mV. However, the exposure to persulfate, bromate and acid breakers did cause a change compared to the base case indicating interactions with polymeric clay stabilizer 1.

The zeta potential values of the samples containing illite fine particles suspended in a solution of distilled water and polymeric clay stabilizer 2 with persulfate, bromate, acid, or enzyme gel breakers after aging at 200–300 °F were 20, 13.91, 4.36, and −13.61 mV, respectively. A similar observation can be seen here as well, the enzyme breaker did not produce a significant change in zeta potential compared to the base case at 300 °F, which had a value of −15 mV. Whereas, the persulfate, bromate and acid breakers produced significant changes, indicating interactions with polymer clay stabilizer 2.

The zeta potential values of the samples containing illite fine particles suspended in a solution of distilled water and 6 wt.% KCl with persulfate, bromate, acid, and enzyme gel breakers after aging at 200–300 °F were −13.67, −12.57, −15.89, and −13.12 mV, respectively. The results show that the KCl clay stabilizer zeta potential value was relatively unaffected by the addition of the different gel breaker types and remained within the range of the base case at 300 °F, which was −16.68 mV.

This clearly shows us that the polymeric clay stabilizers can be influenced by temperature and the presence of persulfate, bromate, or acid gel breakers. This can reduce the performance and prevent polymeric clay stabilizers from stabilizing clays. The use of the enzyme gel breaker did not change the solutions zeta potential values significantly when comparing the base cases at 300 °F for both polymeric clay stabilizers. This was due to the enzymes targeting specific bonds in guar and its derivatives and not interfering with other polymeric additives used.

### 3.4. Polymeric Clay Stabilizer-Gel Breaker GPC Analysis

**Figure 15:**
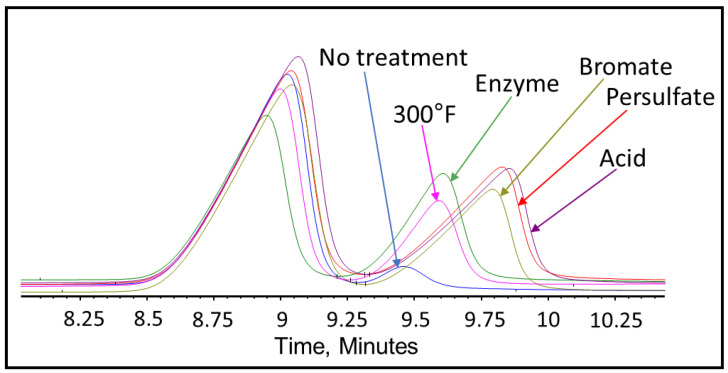
GPC results of polymeric clay stabilizer 1 exposed to different gel breakers (persulfate: 8 ppt, bromate: 8 ppt, acid: 2 gpt, enzyme: 5 gpt) after 24 h at 200–300 °F.

Further analysis was conducted using GPC to determine if the gel breakers had broken any of the polymeric clay stabilizers. Polymeric clay stabilizer 1 and 2 samples were aged using HP/HT aging cells for 24 h. The GPC results for the polymeric clay stabilizer 1 with persulfate at 200 °F, as well as bromate, acid, enzyme gel breakers, and without any gel breaker at 300 °F, are shown in Figure 15. The peak molecular weights of polymeric clay stabilizer 1 are shown in Table 4.

The results show that polymeric clay stabilizer 1 is not significantly affected by temperature due to the small decrease in peak molecular weight. It also shows that the acid had the most effect in reducing the polymeric clay stabilizer size by 30%.

**Figure 16:**
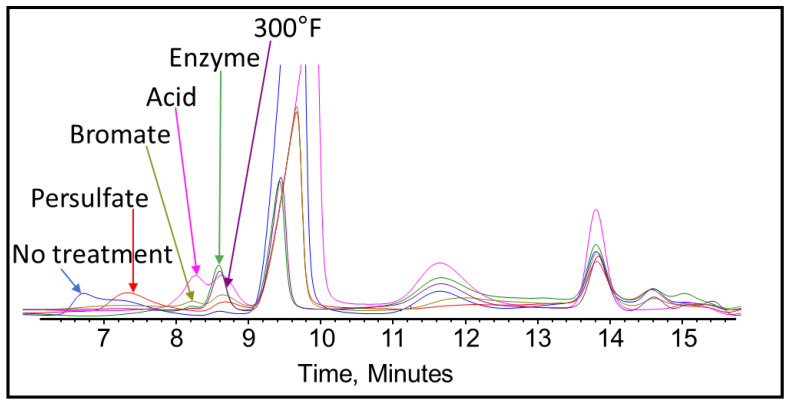
GPC results of polymeric clay stabilizer 2 exposed to different gel breakers (persulfate: 8 ppt, bromate: 8 ppt, acid: 2 gpt, enzyme: 5 gpt) after 24 h at 200–300 °F.

Polymeric clay stabilizer 2 underwent the same gel breaker test as polymeric clay stabilizer 1, and the results for the GPC tests can be found in Figure 16. The peak molecular weight of polymeric clay stabilizer 2 after each test can be found in Table 5.

The results show that temperature had the most effect on the reduction in the molecular weight of polymeric clay stabilizer 2, reducing it by 90%. This is significantly higher than the reduction in peak molecular weight in polymeric clay stabilizer 1. The presence of gel breakers also affects the extent of the reduction of the molecular size. However, in this case, it appears that polymeric clay stabilizer 2 is much more susceptible to temperature effects since at 200 °F with persulfate, the molecular weight decreased the least at 56%.

These changes in molecular weight clearly caused significant changes in zeta potential values and the ability to stabilize clays. These tests show the importance of determining the effect of gel breakers and temperature on the performance of polymeric clay stabilizers prior to any field treatment.

### 3.5. H_2_S-Gel Breaker Interactions

**Figure 17:**
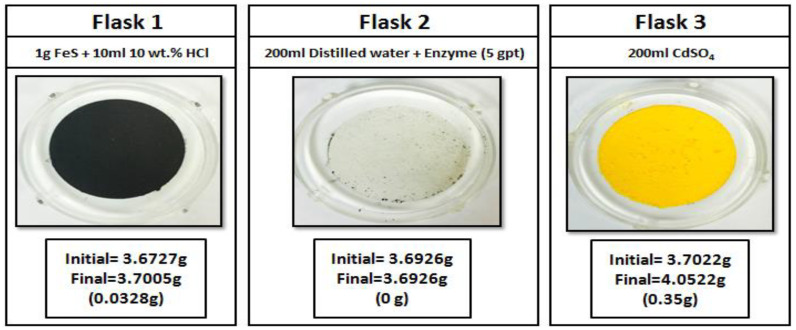
Enzyme gel breaker interactions in a sour environment for 4 h at 77 °F.

**Figure 18:**
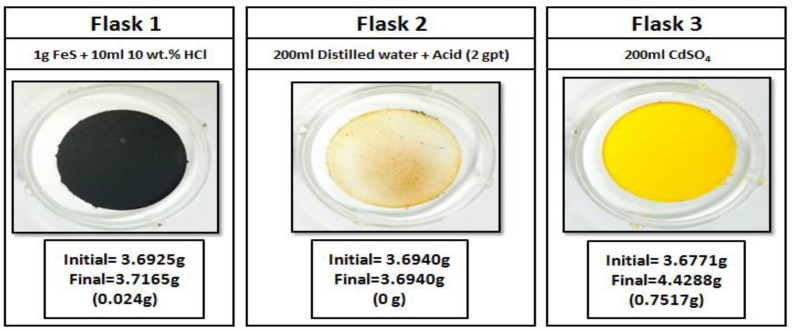
Acid gel breaker interactions in a sour environment for 4 h at 77 °F.

**Figure 19:**
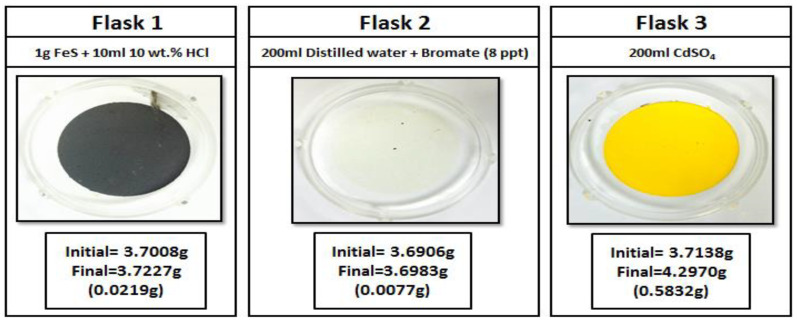
Bromate gel breaker interactions in a sour environment for 4 h at 77 °F.

**Figure 20:**
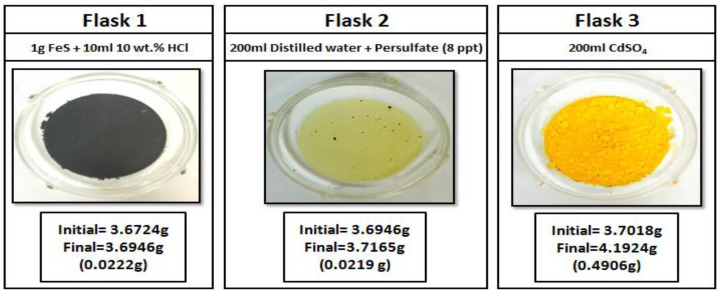
Persulfate gel breaker interactions in a sour environment for 4 h at 77 °F.

Sour environments are common in many formations throughout the world. In addition, some additives such as sodium thiosulfate (commonly used as a high-temperature stabilizer) can generate H_2_S at high-temperature conditions [67]. For that reason, checking additive compatibility with H_2_S becomes important. Different types of gel breakers were analyzed to see if they had any negative interactions in the presence of H_2_S. Enzyme and acid gel breakers showed no precipitation when exposed to H_2_S gas, Figure 17 and Figure 18. On the other hand, bromate and persulfate gel breakers showed precipitation when exposed to H_2_S gas, Figure 19 and Figure 20.

**Figure 21:**
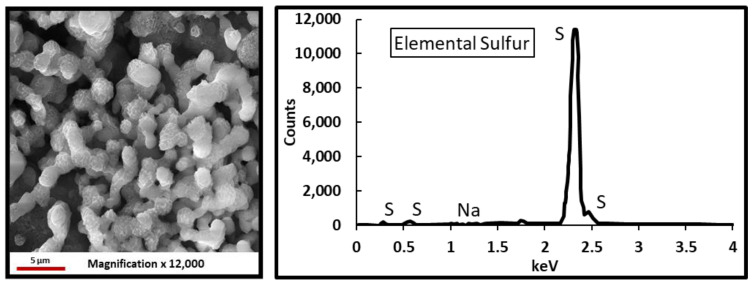
Environmental scanning electron microscope (ESEM) analysis of the precipitation from oxidizer gel breaker interactions in a sour environment for 4 h at 77 °F.

H_2_S can form a variety of species such as sulfate, sulfite, thiosulfate, and elemental sulfur in the presence of oxidants [68,69,70,71]. The precipitation previously seen in flasks 2 were later analyzed using the ESEM/EDS. The results of the ESEM/EDS showed elemental sulfur as the main precipitation, Figure 21. This is due to the oxidizer’s reaction with H_2_S that produces insoluble elemental sulfur, as seen in Equation (1). The amount of sulfur was higher when persulfate gel breakers were used compared to bromate gel breakers.

8H_2_S + 4O_2_ → S_8_ + 8H_2_O (1)

## 4. Conclusions

This work utilized gel permeation chromatography to measure the size of broken polymer fragments to assess the effectiveness of each gel breaker type used in fracturing fluids. The influence of gel breakers on polymeric clay stabilizers and the interactions between gel breaker and H_2_S were also evaluated.

From the lab tests, we can conclude the following:

1. The tested gel breakers were all effective in lowering the viscosity of the 45 lb/1000 gal crosslinked fracturing fluid at 300 °F.
2. The amount of visual polymer residue generated from the use of oxidizer and acid gel breakers is significant and may cause damage to the fracture conductivity.
3. The bromate gel breaker’s intended reactions stopped after 4 h at 300 °F as the broken fracturing fluid polymer size remained constant.
4. Enzyme gel breakers took a longer duration to operate fully; however, they generated the smallest broken polymer fragments and the least residue in comparison to oxidizers and acid gel breakers after 24 h at 300 °F.
5. Heat (300 °F) and gel breakers (acid, bromate) contributed to the break of polymeric clays stabilizers used in this work, the reduction in the size of polymeric clay stabilizers has negatively influenced its performance, which was evidenced by zeta potential measurements.
6. Elemental sulfur precipitation was observed when oxidizers were exposed to H_2_S.

## 5. Recommendations

The authors highly recommend utilizing enzymes to break gelling polymers in fracturing fluids. Enzymes will continue to operate, generating the smallest polymer fragments which correlate directly to reducing damage in the proppant pack, fracture face, and the formation. In addition, enzymes are bond-specific and will not interact with other polymeric additives. The authors also recommend utilizing GPC for polymer-gel breaker analysis. The data obtained from GPC will help in the optimization of time, temperature, gel breaker concentration, and polymer loading for the evaluation of each gel breaker used in field treatments.

## Figures and Tables

**Table 1:** Fracturing fluid formula.

Chemical	Concentration
Polymer (CMHPG)	45 ppt
High-temperature stabilizer (Sodium Thiosulfate)	9 gpt
Zr-crosslinker (Zirconium Triethanolamine)	0.8 gpt
B-crosslinker (Potassium Metaborate)	0.1 gpt

**Table 2:** Used additives composition.

Additive	Main Component
Acid breaker	Chlorous acid
Bromate gel breaker	Sodium bromate
Persulfate gel breaker	Diammonium peroxidisulfate
Enzyme gel breaker	Mixture of 1,6-α-D-galactosidase and endo-1,4-β-mannosidase
Polymeric clay stabilizer 1	Hydroxyalkyl alkylammonium chloride
Polymeric clay stabilizer 2	Polyquaternary amine
Salt clay stabilizer	KCl

**Table 3:** Peak molecular weight values of fracturing fluid polymer with gel breaker samples.

Sample, Concentration	Mp 0.5 Hours (g/mol)	Mp 2 Hours (g/mol)	Mp 4 Hours (g/mol)	Mp 24 Hours (g/mol)
Persulfate gel breaker (8 ppt) @200 °F	1,518,323	501,571	516,813	244,990
Bromate gel breaker (8 ppt) @300 °F	>2,350,000	>2,350,000	386,069	383,142
Acid gel breaker (2 gpt) @300 °F	>2,350,000	>2,350,000	1,142,500	458,188
Enzyme gel breaker (5 gpt) @300 °F	234,276	248,669	1,651,134	12,428
Hydrated Polymer @77 °F	>2,350,000			

**Table 4:** Peak molecular weight values of polymeric clay stabilizer 1 with gel breaker samples after 24 h.

Samples	Peak Molecular Weight (Mp) (g/mol)
PCS1 @ 77 °F	5786
PCS1 + Heat @300 °F	5085
PCS1 + Enzyme gel breaker (5 gpt) @ 300 °F	4950
PCS1 + Bromate gel breaker (8 ppt) @ 300 °F	4241
PCS1 + Persulfate gel breaker (8 ppt) @ 200 °F	4130
PCS1 + 2 gpt Acid gel breaker (2 gpt) @ 300 °F	4021

**Table 5:** Peak molecular weight values of polymeric clay stabilizer 2 with gel breaker samples after 24 h.

Samples	Peak Molecular Weight (Mp) (g/mol)
PSC2 @ 77 °F	107,594
PSC2 + Persulfate gel breaker (8 ppt) @ 200 °F	46,810
PSC2 + Bromate gel breaker (8 ppt) @ 300 °F	17,011
PSC2 + Acid gel breaker (2 gpt) @ 300 °F	16,101
PSC2 + Enzyme gel breaker (5 gpt) @ 300 °F	11,691
PSC2 + Heat @300 °F	11,519

## Nomenclature

µm	Micrometer.
Å	Ampere.
CdSO_4_	Cadmium sulfate.
CMHPG	Carboxymethyl hydroxypropyl guar.
DI	Distilled water.
EDS	Energy dispersive x-ray spectroscopy.
ESEM	Environmentally scanning electron microscope.
FeS	Iron sulfide.
GPC	Gel permeation chromatography.
gpt	Gallons per thousand gallons.
H_2_S	Hydrogen sulfide.
HP/HT	High-pressure/high-temperature.
HPG	Hydroxypropyl guar.
MBI	Monoborate ions.
mm	Millimeter.
Mp	Peak molecular weight.
PDI	Polydispersity index.
ppm	Parts per million.
ppt	Pounds per thousand gallons.
PSC1	Polymeric clay stabilizer 1.
PSC2	Polymeric clay stabilizer 2.
Room temperature	77 °F.
